# “It kinda helped us to be there”: students’ perspectives on the use of virtual patient software in psychiatry posting

**DOI:** 10.1186/s12909-023-04834-9

**Published:** 2023-11-09

**Authors:** Luke Sy-Cherng Woon, Tuti Iryani Mohd Daud, Seng Fah Tong

**Affiliations:** 1 Department of Psychiatry, Faculty of Medicine, Universiti Kebangsaan Malaysia, Cheras, Kuala Lumpur Malaysia; 2 Department of Family Medicine, Faculty of Medicine, Universiti Kebangsaan Malaysia, Cheras, Kuala Lumpur Malaysia

**Keywords:** Clinical clerkship, Experience, Medical students, Psychiatry, Virtual patients

## Abstract

**Background:**

At the Faculty of Medicine of the National University of Malaysia, a virtual patient software program, DxR Clinician, was utilised for the teaching of neurocognitive disorder topics during the psychiatry posting of undergraduate medical students in a modified team-based learning (TBL) module. This study aimed to explore medical students’ learning experiences with virtual patient.

**Methods:**

Ten students who previously underwent the learning module were recruited through purposive sampling. The inclusion criteria were: (a) Fourth-year medical students; and (b) Completed psychiatry posting with the new module. Students who dropped out or were unable to participate in data collection were excluded. Two online focus group discussions (FGDs) with five participants each were conducted by an independent facilitator, guided by a questioning route. The data were transcribed verbatim and coded using the thematic analysis approach to identify themes.

**Results:**

Three main themes of their learning experience were identified: (1) fulfilment of the desired pedagogy (2), realism of the clinical case, and (3) ease of use related to technical settings. The pedagogy theme was further divided into the following subthemes: level of entry for students, flexibility of presentation of content, provision of learning guidance, collaboration with peers, provision of feedback, and assessment of performance. The realism theme had two subthemes: how much the virtual patient experience mimicked an actual patient and how much the case scenario reflected real conditions in the Malaysian context. The technical setting theme entailed two subthemes: access to the software and appearance of the user interface. The study findings are considered in the light of learning formats, pedagogical and learning theories, and technological frameworks.

**Conclusions:**

The findings shed light on both positive and negative aspects of using virtual patients for medical students’ psychiatry posting, which opens room for further improvement of their usage in undergraduate psychiatry education.

**Supplementary Information:**

The online version contains supplementary material available at 10.1186/s12909-023-04834-9.

## Background

The COVID-19 pandemic and the ensuing lockdown measures highlight the need for the incorporation of online learning in medical education. Among other tools, virtual patient software can be useful in teaching medical students across various clinical disciplines. While the value of virtual patient software in undergraduate psychiatry education is gaining recognition [1], understanding of medical students’ experiences with their use is still limited. Such understanding may help in the full utilisation of this educational tool to provide valuable learning opportunities for students.

The term “virtual patients” has been used to describe various applications of information technology for clinical teaching. A review shows that interactive patient scenarios have been the predominant type of virtual patient in medical education [2]. This is in line with the American Association of Medical Colleges’ definition that virtual patients involves simulation of real-life clinical scenarios using computer based programs [3]. Other common types of virtual patients are high fidelity software simulations and virtual standardised patients [2]. Comparing to other forms of virtual patients that emphasise different competencies, such as procedural skills or communication skills, interactive patient scenarios gravitate towards supporting the development of student clinical reasoning skills [4].

Earlier studies have favourably compared the use of virtual patient software with standardised patients and real patients in undergraduate medical education [5]. Students find it helpful that virtual patients can demonstrate clinical abnormalities, and there is a high level of acceptance [6]. It may also be useful for early medical students with less clinical experience, as they may encounter difficulty in communication and history-taking with real or standardised patients [7]. A recent systematic review shows that the use of virtual patients helps improve students’ clinical reasoning skills, especially in data collection, diagnosis, and patient management [8]. This agrees with the values of virtual patient simulation in improving skills and knowledge in wider health education, including other allied professions [9].

Students’ perceptions of virtual patient software have also been assessed in some qualitative studies. Students experience interactions with virtual patients as integrating biomedical knowledge and clinical experience, which provide learning in a logical and structured process. Nonetheless, the virtual patients were also perceived as monotonous, lacking in emotional interactivity and complexity of real patients [10, 11]. Besides the issue of authenticity, the adequacy of feedback in virtual patient tools has also been questioned [12, 13].

Virtual patients are a comparatively novel approach in psychiatry education as opposed to medical education as a whole. Nonetheless, considerable quantitative evidence has been accumulated. In a systematic review and meta-analysis involving 27 randomised controlled trials of the use of simulation in psychiatry for medical doctors [14], significant benefits in the improvement of students’ attitudes, skills, knowledge, and behaviours have been demonstrated, indicating the effectiveness of simulation training in psychiatry education.

Focusing on undergraduate psychiatry education, including medical students and other allied healthcare professionals, a recent systematic review identified 46 articles [1]. Interactive virtual patient scenarios were among the common types of virtual patient interventions. All included controlled trials reported statistically significant positive outcomes in terms of knowledge, skills, and attitudes. Notably, there were only five qualitative studies that attempted to explore students’ learning experiences with virtual patients, indicating a gap in this research area. There were two studies involved nursing students learning using clinical simulation tools [13, 15]. Another mixed methods study of virtual patient simulations focused on Masters level behavioural science students [16]. The remaining two studies examined Danish medical students’ experience with video-based learning in psychiatry using recordings of standardised patients [17, 18]. Hence, in the current literature there is still very limited qualitative research on undergraduate medical students’ psychiatry learning experience with virtual patient tools based on interactive patient scenarios.

At the Faculty of Medicine, the National University of Malaysia, we pioneered the use of DxR Clinician, a virtual patient software program based on interactive patient scenarios, for psychiatry education of fourth-year undergraduate medical students. The students had a seven-week psychiatry posting, during which they were exposed to the discipline through various teaching and learning methods, including ward rounds and clinic sit-ins, online, in-person and hybrid lectures, team-based learning (TBL), and workshops. One of them was a TBL module on neurocognitive disorders, which covered dementia and delirium. The TBL module consisted of the following six steps: pre-class preparation, individual readiness assurance test (IRAT), team readiness assurance test (TRAT), immediate feedback, clinical problem-solving activity, and closing [19]. It was originally designed as a face-to-face session except for the pre-class preparation step. An initiative was taken in the first year of the pandemic (2020) to use DxR Clinician for the teaching of this module. In the modified module, two main changes were made: (1) A virtual interactive case scenario of dementia in DxR Clinician was used for the clinical problem-solving activity that was conducted asynchronously; (2) Other steps that were previously run face-to-face were switched to a videoconferencing platform.

As the subjective experience of students with virtual patients may greatly vary depending on the educational setup and user profile, it is crucial to gain qualitative understanding of students’ experiences within specific contexts. Insights gained from a particular program may in turn inform improvements to current and future efforts in this area. In this study we aimed to explore medical students’ experiences in using the DxR Clinician program for a modified TBL module for neurocognitive disorders during their psychiatry clinical posting at the National University of Malaysia. The SPIDER (sample-phenomenon of interest-design-evaluation-research type) tool was used to frame the research question of this study [20]: “What are the medical students’ experiences of participating in the modified TBL module for neurocognitive disorders using DxR Clinician?” More specifically, we aimed to explore the students’ ideas about and expectations, perceived advantages, and disadvantages of, and perceived barriers to the virtual patient software program in helping them to achieve the objectives of the modified TBL module.

## Methods

The reporting of this study is guided by the Consolidated criteria for reporting qualitative studies (COREQ): a 32-item checklist for interviews and focus groups [21].

### The research team and reflexivity

The research team consisted of three clinical academics (One female and two males) at the Faculty of Medicine of the National University of Malaysia who were involved in undergraduate medical education. Two researchers were psychiatrists (L.S.-C.W. and T.I.M.D.) and another was a general physician (S.F.T.). L.S.-C.W. was the medical lecturer responsible for the modified TBL module for neurocognitive disorders. Both S.F.T and T.I.M.D. had extensive previous experience in conducting qualitative studies. L.S.-C.W. and T.I.M.D. were involved in the clinical teaching of the medical students who took part in this study during their psychiatry posting, but they were not individual supervisors of these students. To avoid bias and undue influence, a PhD student who was not part of this research team was appointed as the facilitator for the data collection process.

### The modified TBL module for neurocognitive disorders

DxR Clinician is a web-based patient simulation software that contains a collection of problem-based case studies for the teaching and evaluating of clinical reasoning skills of medical students. It allows the student to interview the virtual patient, conduct a simulated examination, and order lab tests [22]. A wide range of interview questions (up to 250 questions) is included for history taking. The software is also able to simulate physical exams and provide interpretation of findings, besides allowing the ordering of laboratory tests and imaging studies. Based on the findings, the students can develop a list of working hypotheses and follow the evidence to decide the final diagnosis. Finally, a treatment plan can be developed. Immediately after the completion of the case study, performance feedback in the form of a composite score with a breakdown of different component scores is provided to the students to indicate their strengths and weaknesses.

Using DxR Clinician, a modified TBL module for neurocognitive disorders was developed. The learning objectives were to: define the condition; describe and define relevant symptoms and signs; discuss relevant differential diagnoses; discuss relevant investigations; and discuss principles of management. A virtual case in DxR Clinician was prepared to present a clinical scenario with the required primary psychiatric condition and associated secondary features that would stimulate students’ critical thinking about differential diagnoses. To ensure the authenticity of the case scenario, the virtual case was adapted from an actual case write-up for a patient with dementia, but with alterations of clinical features and modifications of sociodemographic characteristics to ensure anonymity.

The class was conducted in three phases. In Phase 1, pre-reading materials and video instructions for the use of DxR Clinician were provided to the students a few days before the class. In Phase 2, an online session using a video-conferencing platform was held. An online quiz was administered to the students individually, followed by answering the quiz in groups and discussing the quiz questions. This formed the individual and group readiness assurance tests, respectively, for TBL. The student groups were then instructed to begin their case study using DxR Clinician (Phase 3). This section of the learning process was done asynchronously, whereby the students worked through the case with their group members. Students were expected to arrange the learning activity at this phase with their peers, including the time, place, and device to be used. As the case study was conducted in groups, each student group was given a group account name and a password to access the web-based program and solve the case. A final debriefing session was held after that, during which the instructor provided feedback, comments, and further instructions regarding the students’ case-based learning activity.

### Study design

A qualitative study design was chosen for this research. A qualitative design is suitable to answer the research question regarding study subjects’ perceptions and experiences, as it allows a more in-depth and holistic understanding of phenomena within the natural context of the subjects [23]. In this study, we adopted the basic positions of critical realism. Ontologically, we affirmed the mind-independent existence of reality. At the same time, we accepted epistemological constructivism, that the understanding of reality is a function of our perspectives, and there could be multiple interpretations of reality. We assumed that social reality is driven by mechanisms influenced by the context [24, 25]. However, we did not clearly delineate the context, mechanisms, and outcomes we investigated in this study. While we attempted to provide theoretical explanations for our findings, drawn from other theories, on how virtual patients for psychiatry education of medical students works, we did not develop or test a program theory [25].

### Study population

Medical students at the National University of Malaysia were enrolled in a five-year undergraduate program for the Doctor of Medicine degree (MD). The program consisted of a preclinical phase of basic medical sciences for two years, followed by a junior clinical clerkship in the third year and two more years of senior clerkship. Students enter clinical posting in psychiatry during the fourth year. The inclusion criteria for this study were: (a) Fourth-year medical students; and (b) Completed psychiatry posting with the new modified TBL module for neurocognitive disorders. The exclusion criteria were: (a) Dropped out from studies; (b) Unable to devote time for participation in data collection due to commitment to other activities, physical or mental poor health, or other issues. A total of 128 fourth-year medical students completed the modified TBL module with DxR Clinician at the time this study began.

### Sampling and sample size

Purposive sampling was used to select study participants who were most likely to provide relevant information that would deepen the understanding of the research question [26]. A diverse range of study participants was sampled by including different batches of students who underwent their psychiatry posting at different times (which in turn affected their clinical exposure due to various levels of restrictions on clinical contact at different periods), and students with different levels of mastery of the study subject. A general idea of the mastery of the subject was given by the DxR case study performance scores achieved by the student groups when they underwent the module.

A focus group size of five participants was chosen. Group size may range from three [27] to 12 [28]. We chose a small group to facilitate the elicitation of in-depth insights [29]. As it was expected that a single focus group would be inadequate, we planned for at least two focus group discussions with a total of 10 participants initially.

Once potential participants were identified, invitations were emailed to the students. The email invitations were delivered by a faculty administrative staff and not one of the researchers to avoid any undue influence. As the potential study subjects were medical students, emphasis was made on the fact that their choice to participate or not will not affect their teaching and learning activities as well as assessments. It was also made clear to them that they were not under any pressure to participate against their will. Students who were available and willing to participate provided their informed consent and were included in the study. The study participants’ age ranged between 23 and 24 years old. There were eight females and two males. Data collection and data analysis was conducted as an iterative process, and as data saturation was achieved after the initial focus group discussions [30], no further sampling was done.

### Data collection

The focus group discussions were conducted online using a videoconferencing platform. A postgraduate student independent of the research team, who had training and experience in running focus group discussions, was appointed as the facilitator. The role of the facilitator involved explaining ground rules, introducing the discussion topic, encouraging participation from all group members, and keeping the discussion on track and on time. The discussions were carried out in English. A questioning route [31] was developed to guide the discussions (Supplementary Material). The online discussions were recorded with the permission of the participants. No third party was present during the discussions. The discussions lasted between about one to one-and-a-half hours. Verbatim transcripts of the conversations during the discussions were made. The transcripts were returned to the participants for review. No correction to the transcripts was requested.

### Data analysis

The thematic analytic approach was used for data analysis [32]. Thematic analysis is a qualitative research method that provides a well-structured approach to handling dataset and summarising its key features and is useful in the examination of different perspectives of research participants [32, 33]. The same analytic approach has also been used by other qualitative studies on the student experience of virtual patients in undergraduate psychiatry education [1].

The analysis was conducted according to the six phases described by Braun and Clarke [32]. First, the transcribed collected data was read and re-read by the first author (L.S.-C.W.) to familiarise himself with the entire data. Through this process, the researcher actively searched for meanings and patterns in the data within the context of the study. Initial ideas were noted down. Transcripts were labelled with unique codes, which also included the dates FGD was conducted [34]. Next, initial codes were generated. The researcher systematically worked through the data to code interesting features in the data. Relevant data for each code was collated. Data coding and collation of extracts were performed using Microsoft Excel (Redmond, Washington, USA). After all the data had been coded and collated, the codes were examined and considered for the identification of potential themes. With the help of a second researcher (T.I.M.D.), the initial thematic map was developed in this phase to sort the different codes into main themes and subthemes.

Subsequently, all research team members reviewed the themes together, evaluating both the coherence of collated data for each theme and the validity of individual themes in relation to the entire data set. Once this was done, we refined the definitions of the themes and finalised their labels. We deliberated on the ‘story’ each theme tells and decided its place in the overall ‘story’. The results were shared with the study participants, but no additional feedback was received regarding the findings. In the last phase, we produced a write-up of our findings in the form of an analytic narrative with a selection of compelling data extracts to demonstrate the points.

### Ethics approval

This study received approval from the Research Ethics Committee of the National University of Malaysia (UKM PPI/111/8/JEP-2021-355) on 16 July 2021.

## Results

Three main themes were identified regarding the medical students’ experience of using the DxR Clinician program in the modified TBL format in their psychiatry posting: (1) fulfilling the desired pedagogy (2), realism of the clinical case, and (3) ease of use related to technical settings (Table 1). Participants’ quotations are included in the following subsections to illustrate the themes. Each quotation is identified by a unique code.

**Table 1:** Main themes and subthemes

Main theme	Subtheme
Desired pedagogy	Level of entry for students Flexibility of content presentation Provision of learning guidance Collaboration with peers Provision of feedback Assessment of performance
Realism of the clinical case	Similarity to actual patient Cultural adaptation of the case
Technical settings	Access User interface

### Desired pedagogy

The pedagogy theme was mainly about the respondents’ perception of the methods of the learning process using DxR Clinician. The theme covered the whole process of teaching-learning, whether it suited the levels of entry for students, the authenticity of materials, the delivery, peer learning, and assessments in the following sub-themes:

#### Level of entry for students

This subtheme was about what could be the appropriate levels or stages of learning to utilize DxR. First, some participants observed that this software program is suitable for medical students in their preclinical years in exposing them to clinical skills that are required before they enter their clinical postings. It has been suggested that this program can be used in a clinical skills lab or a problem-based learning module for this category of students as a form of preparation for their later clinical years.

*“DxR Clinician is a good platform to begin for preclinical students, at least for them to know what set of questions they should ask…Or what kind of investigation or physical examinations that’s available.” FGD2-28/9/2021*.

At the same time, the virtual patient software program was also viewed as an excellent tool to compensate for the lack of clinical contact with patients during clinical years. The COVID-19 pandemic was cited as a reason for reduced clinical exposure due to the lockdown measures and learning clinical cases through DxR Clinician to some extent mitigated the shortfalls. This was considered especially pertinent for the neurocognitive disorder module in psychiatry posting because of the smaller number of clinical cases the students could encounter in real person compared to other, more common psychiatric disorders.

*“So, since we were like in the pandemic and especially since my psychiatry posting was affected, I feel like it kinda helped us in order to be there even though we can’t be there physically in the ward, but then we can somehow be involved in the entire history taking and all that.” FGD6-4/10/2021*.

It was further suggested that the virtual cases’ difficulty levels could be specified so that students could gauge their competency levels based on case difficulty.

*“I would appreciate it if there is, err… a way to, umm… help different levels of students… I would like it if there were a platform where it tells us what we should know, at our level, so that we are, we are more targeted? And more, umm…able to study better for our level, a bit.” FGD7-4/10/2021*.

#### Flexibility of content presentation

Regarding the presentation of the learning content, explanatory texts and case summaries provided in the case study were considered helpful by some participants. The way information was presented was flexible to fit the user’s style of learning. They can repeatedly use the content suiting their convenience.

*“After we complete the DxR we are provided with, err… many explanations and all the solutions that, err… the cases that we learn. So, I think with this, umm… it actually helps us a lot because sometimes when we learn we don’t- we do not know what sources are correct, or what answers we should give.” FGD10-28/9/2021*.

At the same time, it has been commented that the explanations given were not organised systematically enough, making it hard to digest the content.

*“It actually has tons of explanations but, umm… I don’t, I’m not sure if it is, err… it is not compiled in a very systematic way, I can say. It is quite messy actually like they just put a lot of important points in many paragraphs…” FGD10-28/9/2021*.

Thus, some felt that the manner of information presented in the virtual case study was passive and that they were just ‘going through motion’ when they took part in the learning activity.

*“I think err… some of us might just do it just to get it done. So, err…we don’t try hard, we don’t work hard for it because umm… we just want to get it done because our lecturer wants it to be done. Yeah.” FGD5-28/9/2021*.

Additionally, the issue of the time limit for questioning was also raised. The current format of delivery of content that did not have a time limit might allow more thorough and careful case exploration, but imposing a time limit might improve the efficiency of the learning process.

*“I would prefer a platform with a limitation of time than a limitation of questions because time would be more of an exam-based format and we are used to that. But giving us a limited list or a number of questions would be very hard for us.” FGD3-28/9/2021*.

Lastly, it was also suggested that virtual patients could be used for the development of other clinical skills, such as case presentations to lecturers based on the virtual patient’s history.

#### Provision of learning guidance

In this regard, the opinions of the participants were again varied. Concerning the comprehensive list of questions to select for history taking, some participants thought that it was a helpful resource in guiding their reasoning in actual clinical situations.

*“I would like to add about the variety of questions that can be chosen inside one of the particular cases. There are a lot of questions that we are asking, we will be asking during history taking and this DxR actually, err… includes all kinds of questions, so it provides a very real situation for us to, err… experience it.” FGD9-4/10/2021*.

However, the cues could confuse them if the response did not go in line with their thinking.

*“It really affects the thinking process because rather than you thinking the questions out for yourself, like from the differential diagnosis or anything that you wanna come out with questions, it’s already displayed there then you just select… certain questions where you phrase it another way and you expect another answer, but it came out with a different answer.” FGD2-28/9/2021*.

Yet some other participants viewed the long list of available questions to ask as a drawback. Sometimes the questions could be redundant; sometimes they were too broad or too specific in scope, making it hard for the students to decide if they were relevant to the case.

*“Although I can see there are still, there are a few err… improvements that still to be made* lah. *I mean like, for… for example for certain diagnosis err… or a certain situation, sometimes err…students want to ask, err… a more specific question or more broad question…” FGD1-28/9/2021*.

The participants also observed that as they explored the case, they were not initially aware that the number and relevance of questions they asked would affect their overall performance score later. They would prefer such limitations to be communicated more clearly from the outset.

#### Collaboration with peers

One aspect of pedagogy in this learning module using DxR Clinician was the team-based learning format. This format of learning, which involved collaboration with peers, was seen as beneficial in eliciting performance from the students. The students had the chance to work in a team and discuss the case together. Through such discussions, different perspectives emerged and contributed to the solving of the case.

*“And err, for err, for this psychiatry posting, we actually did this in a group. So, when I was doing it with my friends, I actually thought that I was discussing it with them, so… It’s somehow a good way for me err… to learn and study together. FGD5-28/9/2021*.

The disadvantage of this learning format was that collaborative work could be more time-consuming.

*“My personal disappointment was because we were doing this as a group, and as a group we were only allowed to use, err… one device, instead of everybody using each device to put in their answers and all. So, I feel like because they were using only one device, it just, it, it keen to take a lot more time, because we had to wait for one person to type and the others to give answers, though it, err… makes our teamwork better but then I feel like it just takes some of our time which could be used for something else.” FGD6-4/10/2021*.

#### Provision of feedback

During the asynchronous learning phase using the virtual patient software, the students appreciated the instant, step-by-step feedback as they were working on the case. The feedback provided served as useful guidance to sharpen their clinical reasoning skills by indicating the level of importance of questioning.

*“And what I love the most, err… because DxR provides err… instant feedback, I can say. Err… we can know what we should ask, and what we should not ask, and yeah, the feedback is really good. FGD8-4/10/2021*.

*“But it does help you imprint questions, like when you see this patient, at least you know these questions, if you pick this you get lower marks in your clinic- (laughs) in your DxR so you’re not gonna do that again, and then you will pick a better question for that.” FGD3-28/9/2021*.

An issue highlighted regarding feedback was the interaction with the instructor in the closing session. While instant feedback as assessment scores for the group learning activity was given, the participants also wanted more opportunities to have personal feedback and to ask questions directly. Indeed, such opportunities were provided in the closing session when the instructor went through the case again with the students, but the online session might not be very conducive for interactions.

*“I think some of us after, maybe after doing the whole exercise we might have questions that are more, for example, if it’s physical we could just ask the doctor any kind of questions and, umm… we’ll get their answer, so I’m not sure if this DxR could do the same as well, err… but this will probably require, err… more personal, err… a person to answer a more personalised questi-err… personalised question, yeah. So, I think if there is that function it will be… greater.” FGD7-4/10/2021*.

#### Assessment of performance

The DxR Clinician was viewed favourably as an assessment tool by the study participants. The assessment scores given at the end of the case study served as helpful feedback in evaluating performance. It was suggested that the software program could potentially be used effectively as an assessment tool in examinations.

*“For the current situation, I think DxR is, from what, from what I see* lah, *from what I can think, it’s more like, err… something like exam-based software. Because, you can use DxR as an exam-based software to replace OSCE* lah, *in case, err… student cannot go to the wards or anything, so this platform is very good to use as an exam, exam, err… platform.” FGD1-28/9/2021*.

In keeping with this idea, some felt that virtual patients can assist students in preparing for assessments.

*“It’s a good backup or at least a good, err… revision I would say to read, at least know what kind of questions that you can ask, although it’s not necessary, that means they give you like a set of questions that you know like oh this kind of question can be asked in different cases.” FGD2-28/9/2021*.

Some, however, believed that it was not sufficient to aid preparation for clinical examinations.

*“When you are too used to that kind of platform, discussions are always going around DxR, and then when it’s during the exams, err… they get too comfortable with you know, a certain format and then they can’t go over again.” FGD3-28/9/2021*.

### Realism of the clinical case

This theme is about how closely the clinical case presented in the learning module in DxR Clinician resembled a real clinical encounter. There are two main aspects to this theme.

#### Similarity to actual patient

The first subtheme is related to the ‘feel’ of interacting with the virtual patient – how much it mimicked an actual patient. The opinion that the virtual clinical case was realistic was based on the features of the program that allow users to perform various clinical activities such as history taking, physical examination, and investigations consistent with what happens in real life.

*“It’s as of like you are really clerking a real patient, err… although you can’t get the feel of it, umm… but for example, there’s even the patient’s profile, the faces and it comes as a whole package, so I think it’s also easily accessible in that sense, where I can just, I just need to go into this website, and there is everything from the lab, everyth- investigations and err… the physical examinations, umm… so, I, I thought it’s very, err… err… wholesome in terms of it’s all in one package, and then there is also good visuals, so err… I like that, yeah.” FGD7-4/10/2021*.

On the other hand, the main criticism of the lack of realism of the case was focused on the way history taking is conducted. The questions were pre-listed and the answers from the virtual patient were in written form, therefore differed considerably from real two-way communication between a clinician and a patient.

*“We don’t have really, umm… real feeling of asking patients, because if we are, umm… asking history from a real patient, we have a two-way communication like can hear their voices and all, but in here we just click the button and the, yeah the sentence will come out, the answer will come out like that, so we don’t have the real feeling of asking the patient.” FGD8-4/10/2021*.

#### Cultural adaptation of the case

Another subtheme was about the case scenario: how much the details presented in the scenario reflected real conditions in the local context. Some participants observed that some changes were made to localise the case history content for the learning module, such as the use of local-sounding personal names.

Nonetheless, it was also pointed out that several terms used in the program were not consistent with the common terminologies applied in Malaysia. Furthermore, it has been suggested that having the virtual patient available for history taking in the Malay language (known as *Bahasa Melayu*), the national language of Malaysia, would make the exercise more realistic as Malay is the language most used by students in their clinical encounters with patients, even though the use of English was also prevalent.

*“I’m just curious if this software is also available in* Bahasa*? Yeah, I’m just curious about that because somehow, err, we are living in Malaysia so in our real setting, we’ll be interviewing err…our patient, and we are, maybe, most of our patients we are using* Bahasa, *err*, Bahasa Melayu. *If they wanna make it more real, I think maybe they can like you know… Innovate it into* Bahasa.*” FGD4-28/9/2021*.

### Technical setting

The technical setting theme revolved around two subthemes: access to the software program and the appearance of the user interface.

#### Access

Regarding access, some participants remarked that in the current format, the DxR Clinician clinical case was only accessible to the students using a one-time account during the limited period given for the modified TBL activity. It was hoped that the clinical case could be made available to the students outside of that learning module so that they could explore the case on their own for learning and practice purposes.

*“Because if you want to access the DXR Clinician, you need to have the password of the particular case, but if it’s possible, or if it’s feasible maybe each of the students can have a particular code or particular account to it, and then when we access, we can access towards a different posting, because I believe that DxR Clinician is quite a good software to practise on. So, during our free time or anything I think we can use it for practise.” FGD2-28/9/2021*.

#### User interface

Several comments were made by the participants regarding the user interface (UI). On the positive side, the simple design of the program, which was readily accessible through an internet browser, made it easy to load without taking up too much internet bandwidth.

*“Umm, it is, it doesn’t use a lot of internet bandwidth…So, it is very quickly loaded. The page… (laughs) very simple, just click and then something appears, so it doesn’t have any complexity……on the website.” FGD3-28/9/2021*.

Moreover, it did not require internal storage and was compatible with desktops, laptops, or handheld devices.

On the other hand, the interface graphic design was deemed rather outdated and therefore unattractive. The case directory page was also be quite cluttered as all cases across various disciplines were listed together. The small button size for item selection could be hard to use, especially when navigating using a device with a small screen. Sometimes, the instructions in the software were too wordy or unclear.

*“All right, as for me I think it’s, it’s more about the UI lah. UI and the appearance of the website. The UI’s err… how to say ah, it’s, it’s like from, a very old computer program? I mean like the button, the appearance of the button, location and all… because there are a lot of err… choices and questions, right? Most of the choices are apparently all on the same pages, and so everything looks quite small lah.” FGD1-28/9/2021*.

## Discussion

In this qualitative study, we explored the experiences of undergraduate medical students participating in a team-based learning module in psychiatry using a virtual patient software program. In two focus group discussions, the students shared their perceptions of this mode of learning from their personal experiences. Following a thematic analysis of the transcribed content, we identified three main themes, namely, fulfilment of the desired pedagogy in this mode of learning, realism of the clinical case in the virtual patient program, and its ease of use concerning technical settings. The pedagogy main theme encompasses several subthemes, which are related to several events that occur in the teaching and learning process. The realism theme involves a personal aspect (how much the virtual patient mimics a real patient) as well as a wider sociocultural context (adaptation to the Malaysian context). Lastly, the technical setting theme consists of access issues and user interface appearance.

The use of virtual patient software programs like DxR Clinician in medical education can be considered as a form of case-based learning. Its interactive nature lends itself well to the concept of active learning, which emphasises active and positive engagement of students in the learning process [35]. It is student-centred instead of teacher-centred, where the teacher functions as a facilitator in the quest to acquire knowledge, rather than as the ultimate authoritative source of knowledge [36]. Therefore, it has the potential to work well with the TBL format, which has a similar focus on fostering active learning [37]. Moreover, as the online case study is done asynchronously between the facilitated sessions with ample time given, it also agrees with the flipped classroom approach, which gives students the freedom to engage themselves and review the learning materials at their own pace [38]. In the current study, this is reflected by the participants’ positive experiences with how the program provides information and guidance based on users’ actions and informs their performance in a stepwise manner, and with the TBL format that allows collaboration with peers. Conversely, the lack of contextualised feedback and the logistics and technical difficulties when participating in this learning module were some of the cited issues that might have hindered a truly efficient and enriching active learning experience.

As highlighted by some of the study participants, the learning module afforded them some sort of clinical exposure to neurocognitive disorders despite the severe restrictions during the pandemic lockdown periods, which greatly hampered their access to wards and clinics during psychiatry posting. This points out the value of learning by simulation in psychiatry education. Simulation allows a more ‘hands-on’ learning experience that is contained in a safe environment [39]. A virtual simulated patient made it possible that our students could learn about the clinical case without the risk of COVID-19 infection at the height of the pandemic. Likewise, our study participants also indicated that such a virtual patient software can be very useful for students in preclinical years, precisely because junior students without adequate clinical skills and confidence can still safely learn a topic through a medium like this. Simulation education has been found to reduce anxiety and boost confidence among medical students, improving their readiness and clinical competency in various clinical scenarios [40, 41]. Further exploration of its potential in this aspect of psychiatry education should be considered.

Virtual patients can be valuable pedagogical models serving as a scaffolding for students to prepare them for their clinical encounters with psychiatric patients [1]. We think that the “desired pedagogy” theme could be viewed and understood from the perspective of the nine events of instruction as proposed by the American educational psychologist, Robert Mills Gagné (1916–2002). The events are: Gain attention, inform learners of objectives, stimulate recall of prior learning, present the content, provide learning guidance, elicit a response, provide feedback, assess performance, and enhance retention and transfer [42]. This model is useful in improving instructional design and student performance in medical education [43, 44]. The identified subthemes in this study appear to correspond to some of the components of this model of instructional design. The flexibility of the content presentation subtheme is related to the event of presenting stimulus or content. As suggested by some of the participants, the learning content needs to be meaningfully organised and explained. Next, the provision of learning guidance was perceived as helpful when students were presented with new content, but it can be improved by making it more streamlined and clearer in boundaries. As students work on the virtual case to reach the diagnosis and produce the management plan, the instructional event of eliciting performance occurs. Working in small groups during the case study can help to produce better practice [45, 46]. Collaborative learning helps enhance medical students’ performance [47, 48]. The provision of feedback was seen as important by the study participants, and it highlights the importance of including timely, quality, and student-focused feedback as an essential element of the learning process [49, 50]. Finally, the design of the virtual patient software allows comprehensive assessment scores to be produced for the student group performance, which are directly tied to the learning objectives of the module. Some authors have also suggested that web-based learning modules in psychiatry can be suitably used for tracking learning outcomes [36, 51].

In their research of contextualised learning in the design of a multimedia learning environment, Herrington and Oliver have proposed an instructional design framework for authentic learning environments [52]. Among the nine elements included in the framework, there are two that are closely mirrored by the “realism of clinical case” theme identified in this study. The first is the requirement that the learning experience provides an authentic context that reflects the way the knowledge will be used in real life. This is seen in the students’ desire that the case study demonstrates adaptation to the local Malaysian context in terms of language, culture, and terminology. Research also suggests that localisation of the technology and software for learning that involves cultural and linguistic adaptation is beneficial and may predict better learning outcomes [53]. Furthermore, the extent the study subjects perceived interactions with the virtual patient mimicked actual interactions with a real patient also corresponds with the other element of the framework by Herrington et al., which is the provision of authentic activities during the learning experience. The activities involving the virtual patient should be well-defined and have real-world relevance. Authenticity is regarded as an important aspect of virtual patients, but technical limitations can be an issue [54]. For instance, a software program like DxR Clinician, which employs a more traditional, textual approach to student-patient interaction, is unable to achieve as high a level of fidelity as tools that utilise more advanced technologies, such as immersive virtual reality (VR) [55].

Medical students tend to have a positive attitude towards the use of electronic resources during their training, including psychiatry clerkship [56, 57]. At the same time, there is a concern about the technical quality of electronic educational resources in psychiatry [57]. This technical aspect was given attention by our study participants. In their Conceptual Framework for E-Learning in Developing Countries, Andersson and Grönlund [53] list individual characteristics, contextual factors, and technological challenges as relevant to the delivery of e-learning. Among the technical issues are access and interface design. It was mentioned by some of our study participants that the virtual patient software’s simple design was very helpful in ensuring easy and reliable access to the online platform. However, the settings of user access were something that the students would want to see improvement in. The challenge of the learning module designer is to find the optimal balance between creating greater flexibility in student access to maximise learning gain and ensuring adequate control and monitoring over its usage within the constraints of the software’s current design. Concerning the students’ impression of the software’s UI, a couple of principles drawn from the cognitive theory of multimedia learning may be applied here [58]. The simple, direct UI used in the software could help minimise extraneous cognitive load caused by unnecessary details or ‘flashy’ features, following the Coherence Principle. Conversely, the monotonous appearance might be associated with a lack of cues that highlight salient material, in contravention of the Signaling Principle. Such understanding can be useful in improving the quality of the learning module.

The present study uniquely explored medical students’ experience with virtual patient software based on an interactive clinical scenario in psychiatry education. Its qualitative study design allowed in-depth understanding of students’ perspectives. Several insights gained from this study may inform future research. Among others, strategies to effectively provide feedback to learners in an engaging manner using virtual patients should be further developed and tested. The potential of simulation with virtual patients in psychiatry education in relation to enhancement of clinical competency, especially soft skills, need to be explored. The use of web-based learning modules for the tracking of learning outcomes in psychiatry education also needs more research. Additionally, clearer guidelines are needed for the contextualisation of virtual patients, considering sociocultural differences, students’ technology savviness and available resources, as well as the unique features of the clinical practice of psychiatry in contrast to other medical disciplines.

A few study limitations should be mentioned. We did not collect information from other students who were invited but declined to join the study, therefore we do not know their reasons and whether there was a difference in the characteristics of students who participated and those who did not. While we have tried to make the data collection process more neutral with an independent facilitator for the sessions, the pre-existing student-lecturer relationships between the participants and the researchers might still have influenced the way they responded and expressed their views during the discussions. The participants were in the same clinical year but had their psychiatry posting in several batches over the academic year, thus the time of their last exposure to the learning module varied and they might differ in their ability to recall and share their experiences.

## Conclusions

Our findings suggest that the use of virtual patient software in psychiatry training for medical students has both advantages and disadvantages. From the pedagogical point of view, virtual patients the capability to offer a greater level of flexibility in catering to the needs of students at different levels of competency and with diverse learning objectives. At the same time, careful editing of the case study and related learning materials and instructional guides is required to ensure ease of navigation and use, as well as to maintain students’ focus and motivation to learn. A good match between the learning tool (virtual patient software) and the delivery format is crucial. Low technical specifications may improve learners’ access, but further refinement of the program’s features might be required to meet learners’ expectations of enhanced visual and interactive experience. The use of virtual patient software in undergraduate psychiatry education should be further explored.

### Electronic supplementary material

Below is the link to the electronic supplementary material.

Supplementary Material 1

## Data Availability

The datasets generated and/or analysed during the current study are not publicly available to protect students’ privacy but are available from the corresponding author upon reasonable request.
