# Supplementary material for: “It kinda helped us to be there”: students’ perspectives on the use of virtual patient software in psychiatry posting
Source: BMC Med Educ. 2023 Nov 9;23:851. doi: 10.1186/s12909-023-04834-9 (PMC10636819; doi:10.1186/s12909-023-04834-9)
Supplement: Supplementary file 1 — Supplementary Material 1 [file 12909_2023_4834_MOESM1_ESM.pdf]

<u>Appendix</u>

<u>Questioning Route for the Study</u>

(1) Welcome

- Introduce facilitator

(2) Overview of the topic

- Our topic is…

(3) Ground rules (virtual)

- The session will be recorded
- No right or wrong answers, only differing points of view
- You don't need to agree with others, but you must listen respectfully as others share their views
- My role as moderator will be to guide the discussion
- You are encouraged to talk to each other
- Talk one at a time, mute your microphone when you are not talking
- You can use the ‘raise hand’ function that you wish to talk next
- You are allowed to choose whether to use your real name or remain anonymous
- You are allowed to choose whether to keep your camera on or off

(4) First question

- What do you think about the DxR virtual patient software?

(5) Following questions

- Tell me about positive experiences you've had with the DxR virtual patient software?
- Tell me about disappointments you've had with the DxR virtual patient software?

- How do you feel about DxR virtual patient replacing real patients? *For groups with exposure to real patients
- What went particularly well?
- What needs improvement?
- Suppose that you were in charge and could make one change that would make the class better. What would you do?
- How does it affect your learning/thinking process in arriving at clinical decisions?
- By having a list of questions displayed to you, does it disrupt/affect your thinking process?
- What do you think about the technical aspect of DxR?

(6) Ending questions

- Of all the things we discussed, what to you is the most important?
- Have we missed anything?
